# Supplementary material for: Effects of Proton Pump Inhibitors on Patient Survival in Patients Undergoing Maintenance Hemodialysis
Source: J Clin Med. 2023 Jul 18;12(14):4749. doi: 10.3390/jcm12144749 (PMC10381393; doi:10.3390/jcm12144749)
Supplement: Supplementary file 1 [file jcm-12-04749-s001.zip › jcm-2442764-supplementary.pdf]

Supplementary Material

Table S1. Medication types and Health Insurance Review and Assessment Service codes

Table S2. Cox regression analyses for patient survival using subgroups

Table S3. Patient clinical characteristics after weighting

Figure S1. Study flowchart

Figure S2. Propensity score balance assessment using absolute standardized difference plots

Figure S3. Kaplan–Meier curves of patient survival by group, weighted for confounding factors.

Table S1. Medication types and Health Insurance Review and Assessment Service codes

Medications	Codes
**Acepril**	104201ATB, 104202ATB
**Amlodipine**	495901ATB, 459802ACH, 483201ATB, 486501ATB, 107601ATB, 107601ATD, 459801ACH, 459801ATB, 459901ATB, 464601ATB, 470801ATB, 476201ATB, 479701ATB, 483202ATB, 486502ATB, 107602ATB, 107602ATD, 470802ATB
**Amlodipine+Atorvastatin**	614500ATB, 472300ATB472400ATB472500ATB518900ATB
**Amlodipine+Losartan+Chlorthalidone**	662800ATB, 662900ATB, 663000ATB
**Amlodipine+Losartan+Rosuvastatin**	663900ATB, 664000ATB, 664100ATB, 664200ATB, 664300ATB, 664400ATB
**Amlodipine+Olmesartan+Rosuvastatin**	677300ATB, 677400ATB, 677500ATB, 677600ATB
**Amlodipine+Rosuvastatin**	673900ATB, 674000ATB, 674100ATB
**Amlodipine+Rosuvastatin+Telmisartan**	671200ATB, 671300ATB, 671400ATB, 671500ATB, 677000ATB, 677100ATB, 671600ATB, 671700ATB
**Amlodipine+Telmisartan+Hydrochlorothiazide**	663500ATB, 663600ATB, 663700ATB, 663800ATB
**Amosulalol**	107901ATB, 107902ATB
**Arotinolol**	110202ATB, 110201ATB
**Atenolol**	483102ATB, 111402ATB, 483101ATB, 111403ATB
**Atenolol+Chlorthalidone**	262100ATB
**Azilsartan**	662401ATB, 662403ATB, 662402ATB
**Azilsartan+Chlorthalidone**	673500ATB, 673600ATB
**Barnidipine**	114003ACH, 114001ACH, 114002ACH
**Benidipine**	115101ATB, 115102ATB, 115104ATB, 115103ATB
**Betaxolol**	116801ATB, 116803ATB
**Bevantolol**	117002ATB, 117001ATB
**Bisoprolol**	117904ATB, 117903ATB, 117902ATB, 117901ATB
**Bisoprolol+Hydrochlorothiazide**	469800ATB, 470000ATB, 469900ATB

**Candesartan**	122601ATB, 122602ATB, 122603ATB
**Candesartan+Amlodipine**	652900ATB, 653000ATB, 653100ATB
**Candesartan+Hydrochlorothiazide**	423700ATB
**Candesartan+Rosuvastatin**	661800ATB, 661900ATB, 673700ATB, 662000ATB, 662100ATB
**Captopril**	122901ATB, 122902ATB, 122903ATB
**Captopril+Hydrochlorothiazide**	262200ATB, 262300ATB
**Carteolol**	124801ATB
**Carvedilol**	125005ATB, 125003ATB, 662201ATB, 125008ACR, 125001ATB, 662202ATB, 125007ACR, 125002ATB, 125006ACR, 125004ACR
**Celiprolol**	129101ATB
**Cilazapril**	133001ATB, 133002ATB, 133003ATB
**Cilnidipine**	133102ATB, 133101ATB
**Clonidine**	136505ATR
**Diltiazem**	145706ATB, 145707ACR, 145707ATR, 145703ACR, 145706ATR, 145707ATB
**Doxazocin**	149101ATB, 149102ATB, 149104ATR, 149103ATB
**Efonidipine**	441202ATB, 441201ATB
**Enalapril**	151603ATB, 151601ATB
**Enalapril+Hydrochlorothiazide**	453700ATB, 440300ATB
**Eprosartan**	429201ATB
**Eprosartan+Hydrochlorothiazide**	460500ATB
**Felodipine**	157503ATR, 157501ATR
**Felodipine+Metoprolol**	262400ATR
**Fimasartan**	515203ATB, 515201ATB, 515202ATB
**Fimasartan+Amlodipine**	651900ATB, 652000ATB, 652700ATB, 652100ATB
**Fimasartan+Hydrochlorothiazide**	522000ATB, 526800ATB
**Fimasartan+Rosuvastatin**	655000ATB, 654900ATB, 654800ATB, 654700ATB, 654600ATB
**Fosinopril**	163501ATB, 163502ATB

**Hydralazine**	170701ATB
**Imidapril**	173402ATB, 173401ATB
**Irbesartan**	177301ATB, 177303ATB
**Irbesartan+Atorvastatin**	524000ATB, 524100ATB, 527100ATB, 527000ATB
**Irbesartan+Hydrochlorothiazide**	385700ATB, 385800ATB, 553800ATB
**Lacidipine**	180301ATB, 180302ATB, 180303ATB
**Lercanidipine**	182001ATB, 182002ATB
**Lisinopril**	184501ATB
**Lisinopril+Hydrochlorothiazide**	499200ATB, 499300ATB
**Losartan**	185701ATB, 185702ATB
**Losartan+Amlodipine**	503000ATB, 637400ATB, 513900ATB, 637500ATB, 502700ATB, 637600ATB
**Losartan+Hydrochlorothiazide**	262500ATB, 486900ATB, 378900ATB
**Manidipine**	188001ATB, 188002ATB
**Metoprolol**	194003ATR, 193802ATB
**Metoprolol+Hydrochlorothiazide**	262600ATB
**Minoxidil**	196102ATB
**Nadolol**	198301ATB
**Nicardipine**	201003ACR, 201002ATB
**Nifedipine**	201407ACS, 201405ATR, 528201ATR, 201409ATR, 528202ATR, 201401ACS, 201401ATB, 201408ATR
**Nimodipine**	201901ATB, 356202ATR, 356203ATR, 356201ATB, 356202ATB
**Nisoldipine**	356202ATR
**Olmesartan**	468502ATB, 468501ATB, 468503ATB, 520902ATB, 520901ATB
**Olmesartan+Amlodipine**	547800ATB, 632800ATB, 500500ATB, 547700ATB, 629500ATB, 631300ATB, 500600ATB, 547900ATB, 632900ATB, 547600ATB, 548000ATB, 582200ATB, 629600ATB, 633000ATB, 547500ATB, 582400ATB, 629400ATB
**Olmesartan+Amlodipine+Hydrochlorothiazide**	519800ATB, 519700ATB, 520100ATB, 520000ATB, 519900ATB

**Olmesartan+Hydrochlorothiazide**	513600ATB, 489100ATB
**Olmesartan+Rosuvastatin**	644200ATB, 644100ATB, 526900ATB, 526300ATB, 526400ATB, 653200ATB, 526500ATB
**Perindopril**	211301ATB, 501601ATB, 211302ATB, 501602ATB
**Perindopril+Indapamide**	556200ATB
**Propranolol**	219901ATB, 219904ATB, 219906ACR, 219905ACR
**Quinapril**	221901ATB
**Ramipril**	222401ATB, 222402ATB, 222404ATB
**Ramipril+Felodipine**	447100ATB, 447200ATB
**Ramipril+Hydrochlorothiazide**	448600ATB, 448700ATB
**Telmisartan**	378801ATB, 378802ATB, 378803ATB
**Telmisartan+Amlodipine**	521200ATB, 511600ATB, 521300ATB, 511700ATB, 521400ATB, 511500ATB, 644800ATB, 623100ATB
**Telmisartan+Hydrochlorothiazide**	443200ATB, 443300ATB, 502600ATB
**Telmisartan+Rosuvastatin**	631600ATB, 629900ATB, 630000ATB, 631700ATB, 630100ATB, 630200ATB
**Temocapril**	235002ATB
**Terazosin**	235501ATB, 235502ATB, 235503ATB, 616501ATB
**Valsartan**	247103ATB, 247101ATB, 247102ATB, 247104ATB
**Valsartan+Amlodipine**	522600ATB, 492900ATB, 522900ATB, 523200ATB, 522700ATB, 492800ATB, 522800ATB, 523000ATB, 523300ATB, 495800ATB, 523100ATB, 523400ATB
**Valsartan+Hydrochlorothiazide**	356400ATB, 442600ATB
**Valsartan+Lercanidipine**	522200ATB, 522300ATB, 522400ATB
**Valsartan+Pitavastatin**	635000ATB, 635200ATB, 634900ATB, 635100ATB
**Valsartan+Rosuvastatin**	629700ATB, 525000ATB, 525200ATB, 629800ATB, 525100ATB, 525300ATB
**Valsartan+Sacubitril**	651401ATB, 651402ATB, 651403ATB
**Verapamil**	247606ATB, 247607ATB, 247603ATR, 247605ATR, 247601ACR
**Zofenopril**	510401ATB, 510402ATB, 510403ATB
**Atorvastatin**	111501ATB, 111502ATB, 111503ATB, 111504ATB, 502201ATB, 502202ATB,

	502203ATB, 502204ATB
**Atorvastatin+Amlodipine**	472300ATB, 472400ATB
**Atorvastatin+Ezetimibe**	633800ATB, 633900ATB, 634800ATB
**Fluvastatin**	162401ACH, 162402ACH, 162403ATR
**Lovastatin**	185801ATB
**Pitavastatin**	470901ATB, 470902ATB, 470903ATB
**Pitavastatin+Fenofibrate**	679300ACH
**Pravastatin**	216601ATB, 216602ATB, 216603ATB, 216604ATB
**Rosuvastatin**	454001ATB, 454002ATD, 454002ATB, 454003ATB, 454003ATD, 454005ATB
**Rosuvastatin+Ezetimibe**	640700ATB, 640800ATB, 640900ATB
**Rosuvastatin+Ezetimibe+Telmisartan**	671400ATB, 671500ATB, 671700ATB
**Simvastatin**	227801ATB, 227802ATB, 227803ATB, 227805ATB, 227806ATB
**Aspirin**	110701ATB, 110702ATB, 110801ATB, 110802ATB, 110902BIJ, 111001ACE, 111001ATB, 111001ATE, 111002ATE, 111003ACE, 111003ATE
**Clopidogrel**	133201ACR, 133201ATB, 133201ATR, 133202ATB, 133203ATR, 506100ATB
**Ticlopidine**	498900ATB, 239201ATB, 239202ATB
**Aspirin+Bethocarbamol**	256800ATB
**Aspirin+Clopidogrel**	517900ACH, 517900ACE, 517900ATE, 667500ACE
**Aspirin+Dipyridamole**	489700ACR
**Dexlansoprozole**	621901ACR, 621902ACR
**Esomeprazole**	509901ACH, 367202ATB, 498002ACH, 367202ACH, 509902ACH, 518000ATB, 367201ATB, 523500ATB, 498001ACH, 527400ATB, 367201ACH, 670700ATB, 459401BIJ
**Ilaprazole**	505501ATE
**Lansoprazole**	181301ACH, 181301ATD, 181301ACE, 181302ACH, 181302ATD, 181302ATE, 181302ACE
**Omeprazole**	204403ATE, 204401ACE, 204401ATE, 664500ATB, 204402ATE, 640200ATB, 204501BIJ
**Pantoprazole**	519202ATB, 519202ATE, 208901ATE, 519201ATB, 208802ATE, 519201ATE,

	208902ATE, 208801ATE, 519203ATE, 656701ATE, 208801BIJ
**Rabeprazole**	222201ATE, 222202ATE
**Deflazacort 6mg**	140801ATB
**Dexamethasone 0.5mg**	141901ATB
**Dexamethasone 0.75mg**	141903ATB
**Betamethasone 0.25mg + d-chlorpheniramine 2mg**	296900ATB
**Hydrocortisone 10mg**	116401ATB
**Hydrocortisone 5mg**	170901 ATB
**Methylprednisolone 4mg**	193302 ATB
**Methylprednisolone 1mg**	193305 ATB
**Prednisolone 5mg**	217001 ATB
**Triamcinolone 1mg**	243201 ATB
**Triamcinolone 2mg**	243202 ATB
**Triamcinolone 4mg**	243203 ATB
**Fludrocortisone 100 µg**	160201 ATB

Table S2. Cox regression analyses for patient survival using subgroups

	Univariate		Multivariate	
	**HR (95% CI)**	***P***	**HR (95% CI)**	***P***
**Patients with coronary artery disease**				
Ref.: Group 1				
Group 2	0.93 (0.76–1.14)	0.506	0.91 (0.70–1.18)	0.477
Group 3	1.00 (0.84–1.20)	0.958	1.09 (0.87–1.38)	0.454
Ref.: Group 2				
Group 3	1.08 (0.84–1.37)	0.552	1.20 (0.89–1.62)	0.232
**Patients without coronary artery disease**				
Ref.: Group 1				
Group 2	1.07 (1.02–1.12)	0.009	0.99 (0.94–1.05)	0.814
Group 3	1.27 (1.22–1.33)	<0.001	1.09 (1.04–1.15)	<0.001
Ref.: Group 2				
Group 3	1.19 (1.13–1.27)	<0.001	1.10 (1.02–1.18)	0.010
**Patients with steroid usage**				
Ref.: Group 1				
Group 2	0.98 (0.79–1.21)	0.832	0.86 (0.66–1.11)	0.243
Group 3	1.27 (1.08–1.49)	0.005	1.05 (0.85–1.29)	0.645
Ref.: Group 2				
Group 3	1.30 (1.02–1.65)	0.032	1.23 (0.92–1.64)	0.171
**Patients without steroid usage**				
Ref.: Group 1				
Group 2	1.08 (1.03–1.29)	0.003	1.00 (0.94–1.06)	0.952
Group 3	1.27 (1.22–1.32)	<0.001	1.09 (1.03–1.15)	<0.001
Ref.: Group 2				
Group 3	1.18 (1.11–1.25)	<0.001	1.09 (1.02–1.17)	0.018

**Patients with gastrointestinal disease**				
Ref.: Group 1				
Group 2	0.97 (0.72–1.32)	0.862	0.78 (0.53–1.14)	0.195
Group 3	1.15 (0.91–1.44)	0.235	0.91 (0.68–1.21)	0.518
Ref.: Group 2				
Group 3	1.18 (0.84–1.66)	0.346	1.17 (0.77–1.79)	0.457
**Patients without gastrointestinal disease**				
Ref.: Group 1				
Group 2	1.07 (1.03–1.13)	0.003	1.00 (0.94–1.06)	0.959
Group 3	1.27 (1.22–1.33)	<0.001	1.10 (1.04–1.15)	<0.001
Ref.: Group 2				
Group 3	1.19 (1.12–1.26)	<0.001	1.10 (1.02–1.18)	0.010
**Patients with antiplatelet agent usage**				
Ref.: Group 1				
Group 2	1.01 (0.94–1.08)	0.840	0.96 (0.87–1.05)	0.346
Group 3	1.22 (1.14–1.30)	<0.001	1.06 (0.98–1.15)	0.164
Ref.: Group 2				
Group 3	1.21 (1.10–1.32)	<0.001	1.11 (0.99–1.24)	0.080
**Patients without antiplatelet agent usage**				
Ref.: Group 1				
Group 2	1.10 (1.04–1.17)	0.002	1.02 (0.95–1.10)	0.539
Group 3	1.26 (1.20–1.33)	<0.001	1.12 (1.06–1.19)	<0.001
Ref.: Group 2				
Group 3	1.14 (1.06–1.23)	<0.001	1.10 (1.00–1.20)	0.040

Multivariate analysis was adjusted for age, sex, underlying cause of end-stage renal disease, Charlson Comorbidity Index score, vascular access type, hemodialysis vintage, ultrafiltration volume, Kt/V_{urea}, hemoglobin, serum albumin, serum creatinine, serum phosphorus, serum calcium, systolic blood pressure, diastolic blood pressure, and use of anti-hypertensive drugs, aspirin, and statins, and was performed using enter mode. Abbreviations: CI, confidence interval; HR, hazard ratio.

Table S3. Patient clinical characteristics after weighting.

	Group 1	Group 2	Group 3	P
Age (years)	60.2 ± 0.1	60.0 ± 0.2	60.6 ± 0.2	0.318
Sex (male, %)	59.9%	58.4%	58.9%	0.159
Hemodialysis vintage (days)	1567 ± 8.5	1562 ± 25	1524 ± 24	0.849
Underlying disease of ESRD				0.182
Diabetes mellitus	44.1%	43.4%	43.3%	
Hypertension	26.2%	26.3%	26.3%	
Glomerulonephritis	10.4%	10.5%	11.9%	
Others	8.4%	8.8%	8.0%	
Unknown	10.8%	11.0%	10.5%	
CCI score	7.5 ± 0.0	7.6 ± 0.0	7.7 ± 0.0	0.001
Type of vascular access				0.554
Arteriovenous fistula	85.2%	84.7%	85.2%	
Arteriovenous graft	14.8%	15.3%	14.8%	
Kt/V_{urea}	1.53 ± 0.00	1.53 ± 0.00	1.53 ± 0.00	0.670
Ultrafiltration volume (L/session)	2.28 ± 0.00	2.26 ± 0.01	2.25 ± 0.01	0.192
Hemoglobin (g/dL)	10.7 ± 0.0	10.7 ± 0.0	10.7 ± 0.0	0.990
Serum albumin (g/dL)	3.99 ± 0.00	3.99 ± 0.00	3.98 ± 0.00	0.289
Serum phosphorus (mg/dL)	5.0 ± 0.0	5.0 ± 0.0	4.9 ± 0.0	0.736
Serum calcium (mg/dL)	8.9 ± 0.0	8.9 ± 0.0	8.9 ± 0.0	0.838
Systolic blood pressure (mmHg)	141 ± 0	141 ± 0	141 ± 0	0.675
Diastolic blood pressure (mmHg)	78 ± 0	78 ± 0	78 ± 0	0.991
Serum creatinine (mg/dL)	9.5 ± 0.0	9.5 ± 0.0	9.5 ± 0.0	0.878
Use of antihypertensive drugs	68.4%	70.8%	72.4%	<0.001
Use of aspirin	42.8%	44.6%	45.7%	0.004
Use of stain	29.4%	31.0%	31.8%	0.006

Data are expressed as mean ± standard error for continuous variables and as percentages for categorical variables. *P*–values were tested using one–way analysis of variance for continuous variables and Pearson's χ^2 test for categorical variables.

Abbreviations: CCI, Charlson comorbidity index; ESRD, end–stage renal disease.

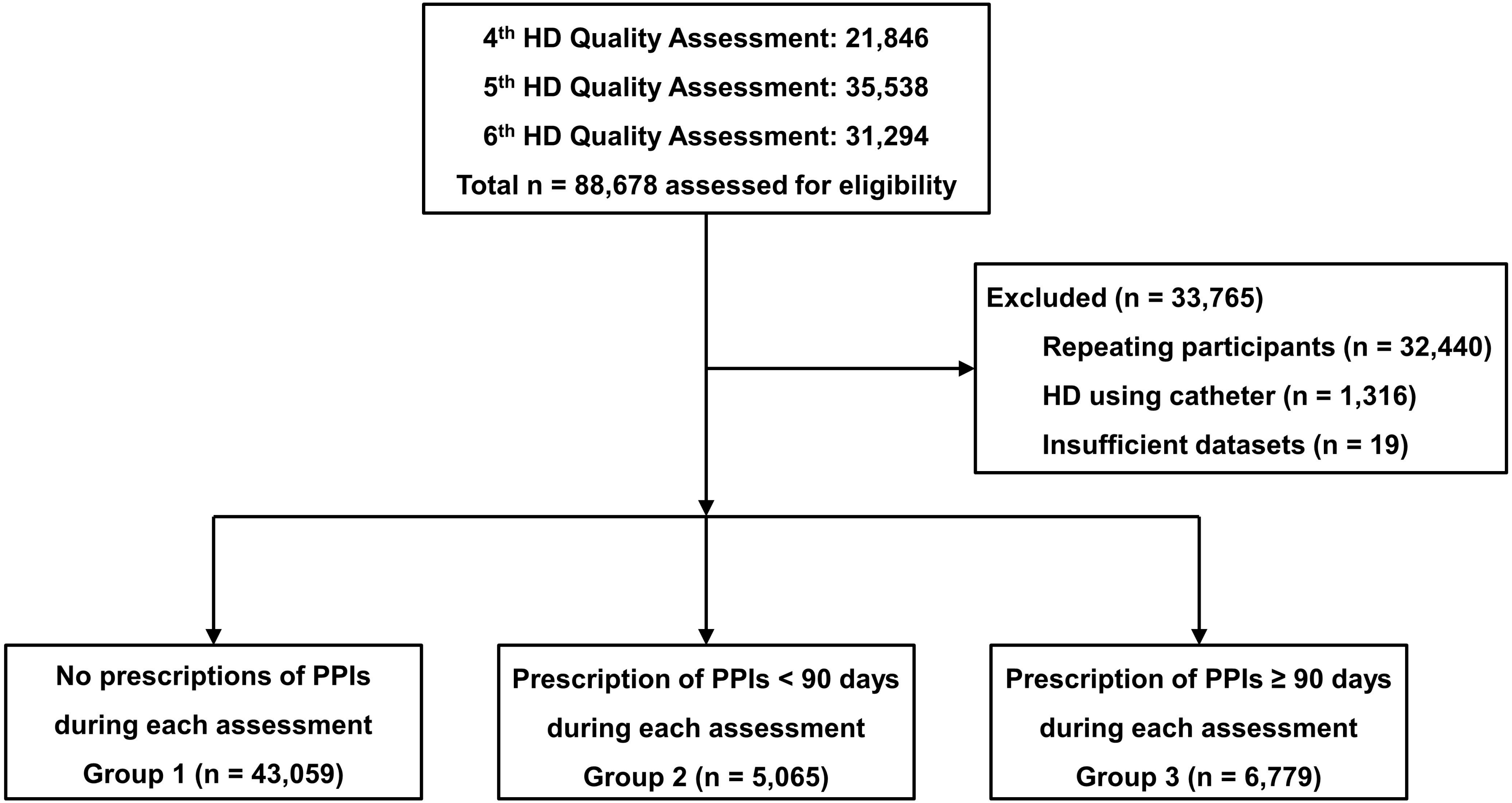

Figure S1. Study flow chart.

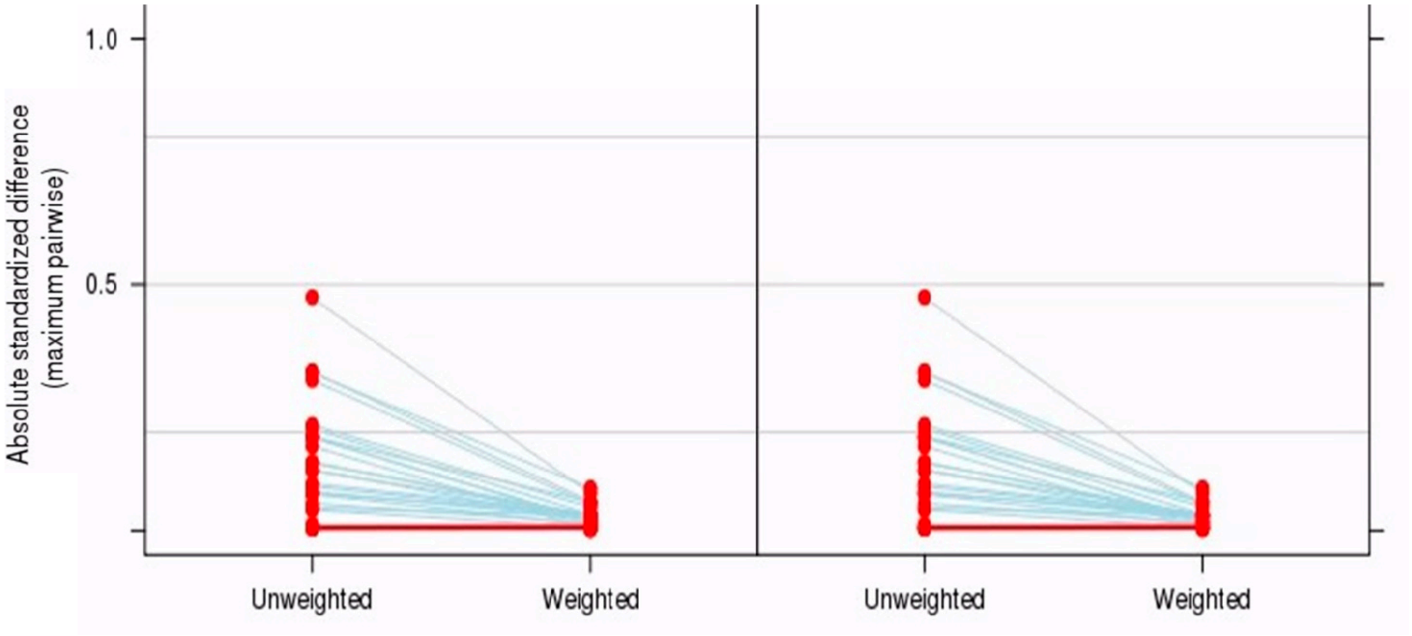

Figure S2. Propensity score balance assessment using absolute standardized difference plot. Left panel: effect size. Right panel: Kolmogorov-Smirnov statistics.

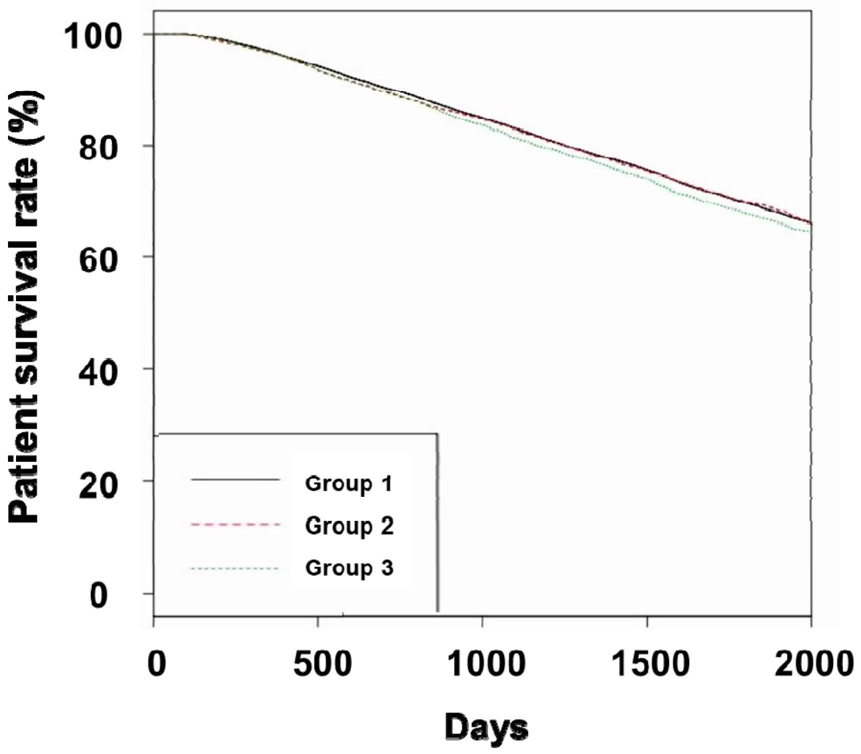

Figure S3. Kaplan–Meier curves of patient survival by group, weighted for confounding factors. The 5–year survival rates in Groups 1, 2, and 3 were 69.2%, 69.6%, and 67.2%, respectively.
